# Necessity of dental restoration before heart valve replacement: a cross-sectional study of a historical cohort

**DOI:** 10.1007/s00784-025-06637-2

**Published:** 2025-11-10

**Authors:** Lucas M. Ritschl, Isabell Bernlochner, Andreas Keßler, Klaus-Dietrich Wolff, Herbert Deppe

**Affiliations:** 1 Department of Oral and Maxillofacial Surgery, TUM University Hospital Klinikum Rechts der Isar, School of Medicine and Health, Technical University of Munich, Ismaninger Strasse 22, Munich, D-81675 Germany; 2 Department of Internal Medicine I (Cardiology, Angiology and Pneumology), TUM University Hospital Klinikum Rechts der Isar, School of Medicine and Health, Technical University of Munich, Munich, Germany; 3 Department of Conservative Dentistry and Periodontology, University Hospital, Ludwig-Maximilians-University Munich, Munich, Germany; 4 Department of Prosthetic Dentistry, Faculty of Medicine, Centre for Dental Medicine, Medical Centre, University of Freiburg, Freiburg, Germany

**Keywords:** Heart valve replacement, Endocarditis, Prosthetic valve infective endocarditis, Dental focus

## Abstract

**Objectives:**

This study aimed to assess the dental status of patients referred for preoperative dental evaluation prior to heart valve replacement, to analyze the consultation process, and to determine the necessity of dental-surgical intervention.

**Materials and methods:**

This retrospective study included only outpatients referred for dental focus screening before heart valve replacement between January 1, 2020, and March 31, 2024.

**Results:**

The 66 patients included had a median age of 68.5 years (33.3–88.4) and comprised 16 women and 50 men. Valve replacements were distributed as follows: 65.2% aortic, 27.3% mitral, 1.5% pulmonary, and 6.0% unknown. In 89.4% of cases, outpatient consultation occurred ≤ 10 days before surgery. The DMFT index was 20.0 (10–28) in aortic and 18.5 (6–28) in mitral valve disease (*p* = 0.209). Patients with dental restorations had a DMFT of 20.0 (12–28), and those without had 17.5 (12–28) (*p* = 0.304). A significant but weak correlation was found between the DMFT index and heart valve disease (ρ = -0.277; *p* = 0.029). In 48.5% (*n* = 32), oral and maxillofacial surgical intervention was indicated. Among patients requiring intervention, 60.5% had aortic and 22.2% mitral valve disease (*p* = 0.007).

**Conclusion:**

Future efforts should prioritize interdisciplinary education and patient engagement to prevent the rare but serious prosthetic valve endocarditis, aiming to reduce adverse outcomes after valve replacement.

**Clinical relevance:**

Given the increasing incidence of prosthetic valve infective endocarditis (PVE), this study highlights the critical role of preoperative dental assessments in identifying potential oral foci. Nearly half of the patients required oral or maxillofacial surgical intervention before heart valve replacement, emphasizing the importance of timely interdisciplinary collaboration. Integrating dental evaluations into the standard preoperative protocol may help reduce postoperative complications and improve patient outcomes.

## Introduction

Demographic trends, lifestyle changes, and other factors are leading to an increase in the incidence of heart valve disease in the population. Accordingly, the number of heart valve replacements is also increasing. Heart valve replacements can be performed either conventionally by open surgery (surgical aortic valve implantation, SAVR) or catheter-supported (transcatheter heart valve replacement). The latter procedure can be carried out either endovascularly (e.g., as transcatheter aortic valve implantation, TAVI) or transapically. In 2022, 38,547 heart valve procedures (biological/mechanical valve replacement or valve reconstruction) were performed in Germany alone, which represents a significant increase compared to 22,243 procedures in 2008 [1].

With the increasing frequency of implantation of artificial heart valves, the rate of prosthetic valve infective endocarditis (PVE) has also risen [2], accounting for 10–34.1% of all cases of infective endocarditis (IE) [2, 3]. PVE is a rare but most severe form of IE, occurring in 1–7.4% of patients with a prosthetic heart valve [4, 5], with an incidence of 0.3–1.9% per 100 patient-years [6] or 2.4–4.9 cases per patient-year [7]. PVE occurs in approximately half of cases within the first year after valve replacement [8] and more commonly affects biological than mechanical prosthetic valves [9]. Early PVE appears to occur more frequently after TAVI than after SAVR [10]. The median time interval between TAVI and PVE was found to be 461 days [11]. Fauchier et al. [12] described in their study a post-TAVI PVE of 398 days versus a post-SAVR PVE of 469 days. Generally, PVE has a significant impact on morbidity and healthcare costs [13]. The most common complication is heart failure, which occurs in 37% of post-TAVI PVE cases [14]. Other complications include acute heart failure, acute renal failure, septic shock, acute myocardial infarction, and systemic embolism [15]. Mortality and stroke risk remain high despite early diagnosis and treatment [16]. The PVE-associated hospital mortality rate and one-year mortality rate are described as 15.2–29.5% [3, 14] and 18.9–42.3% [3, 8], respectively.

In the prospective European infective endocarditis (EURO-ENDO) registry study of the European Society of Cardiology, it was found that the most frequent previous noncardiac procedures performed in the previous six months were of a dental nature (7.9%) in the 3,116 patients included and that the systemic entry was localized in the dental field in 9.8% of cases [2]. In a study by Deppe et al., in which the surgically removed heart valves of 134 patients were examined microbiologically, the oral cavity was identified as the source of pathogens in 10.4% of cases [17]. The oral cavity is colonized by relevant pathogens, including *streptococci* of the oral group, and represents an important portal of entry. Oral *streptococci* include the *mitis*,* sanguis*,* anginosus*,* salivarius*,* downei*, and *mutans* groups [6]. The *mitis* group is currently the largest of the groups found in the oral cavity with 20 species [18]. Oral surgery (including all extractions, periodontal surgery, implant surgery, and oral biopsies) and dental procedures involving manipulation of the gingiva or periapical area of the teeth are considered invasive dental procedures and are at increased risk of bacteremia [6]. Recent publications have confirmed the link between invasive dental procedures and the development of IE, bringing renewed attention to oral hygiene and procedures [19]. The literature indicates that poor oral hygiene increases the risk of IE in people at medium and high cardiac risk. In this patient group with an increased risk of IE, in addition to regular dental checkups, awareness should be raised of professional dental cleaning [20]. Further, as the incidence of IE is generally escalating [21], knowledge of its prevention is essential.

The aims of this study were therefore [1] to critically assess the dental status of the patients presented for a consultative focus search [2], to analyze the treatment process of this consultative procedure, and finally [3] to determine the need for dental-surgical action prior to a heart valve replacement.

## Materials and methods

### Ethical statement and study population

This monocentric, retrospective study complied with the current Helsinki Declaration and was reviewed and approved by the Ethics Committee of the TUM School of Medicine and Health of the Technical University of Munich on April 30, 2024 (approval number 2024-188-S-CB). The data collection for the study covered only patients who presented as outpatients of the German Heart Center Munich (Lazarettstrasse 36, 80636 Munich) at the Department of Oral and Maxillofacial Surgery, TUM University Hospital Klinikum Rechts der Isar, School of Medicine and Health, Technical University of Munich with a request for a dental focus screen prior to heart valve replacement in the period from January 1, 2020, to March 31, 2024.

### Data collection and management

In a first step, all outpatients who had presented for a consultative focus search prior to any intervention (e.g., stem cell transplantation, administration of antiresorptive drugs, organ transplantation) were screened. The general master data as well as data and information specific to the patient’s health and course of illness were recorded from the manually maintained outpatient card and the hospital information system. From this heterogeneous collective, the cardiological patients who specifically presented for consultation with the question “dental focus or dental restoration before heart valve replacement” were included in the next step. From this homogenized collective, the tooth-specific data and heart specific findings were then extracted from the handwritten outpatient clinic cards. Heart specific findings included e.g. heart failure symptoms according to NYHA functional classification (New York Heart Association I–IV). In addition, all – exclusively available – dental radiographs (OPG = orthopantomogram) were reviewed and analyzed with regard to decayed, missing or filled teeth resulting in the DMFT index [22]. The further clinical course was assessed with regard to the need for oral and maxillofacial surgery in the sense of an invasive dental procedure [6, 23], and the number of teeth to be extracted, course (inpatient or outpatient), duration of the operation, incidence of the use of aids to prevent an expected postoperative bleeding complication (dressing plate, Spongostan Standard [hemostatic agent; Johnson & Johnson Services Inc.), and incidence of postoperative complications (bleeding, abscess formation, or wound healing disorder).

In the analysis, all teeth in which there was a reasonable suspicion of a cavity in the dentin layer or which definitely had a cavity were assigned to component D (= decayed), missing teeth to component M (= missing), and filled and crowned teeth to component F (= filled). The DMFT index is determined on the basis of the number of these three findings and generally reflects the degree of caries of the person examined. Regular visits to the dentist were defined as once a year.

In a final step, the timing of the consultation in the overall context of the planned cardiological intervention (time interval between outpatient consultation and planned cardiological intervention [≤ 10 days or > 10 days]; time interval between outpatient consultation and dental surgery [days]) and the quality of communication and data transfer (doctor’s letter, consultation request/sheet, laboratory values) between the two clinics was recorded. The cut-off of the time interval between outpatient consultation and planned cardiologic intervention of ≤ 10 days was justified by the fact that, from a purely surgical point of view, the sutures are removed after ten days once the wound has healed, meaning that oral and maxillofacial surgery would be released for further interventions [24].

### Statistical analyses

All statistical tests were performed with a two-sided significance level of 5%. No adjustments were made for multiple testing. The analysis was performed using IBM SPSS 24 for Mac software (IBM Corp, Armonk, New York, United States). The Mann–Whitney U test was used, for example, in the subgroup analysis (patients with aortic or mitral valve disease). The two-sided bivariate rank correlation coefficient (pairwise case exclusion) according to Spearman (ρ) was used to show the relationship between the DMFT index and cardiac valve disease and the frequency of a need for action.

## Results

During the observation period, a total of 91 cardiological patients with a median age of 66.7 years (24.9–88.4) and a gender distribution of 21 women and 70 men were sent for consultation prior to a planned intervention. Of this group, 66 (72.5%) patients underwent a focal search prior to planned heart valve replacement. In the remaining 25 patients (27.5%), the indication for consultation was distributed as follows: ten for postoperative heart valve endocarditis, eight for dental focus search in current endocarditis, six before planned coronary bypass surgery, and one for unclear infectious focus.

### General medical history

The 66 included patients had a median age of 68.5 years (33.3–88.4) and a gender distribution of 16 women and 50 men. NYHA class II was present in 6.1% and NYHA class III in 1.5% of cases. In 92.4% the NYHA class was unkown. Fifteen patients had diabetes mellitus, of which only one was type 1. Fifteen patients smoked and 44 patients had arterial hypertension, which was treated in all but one case. The usual drugs used to treat arterial hypertension were beta-blockers in 33.3% of patients, combination preparations of beta-blockers and ACE inhibitors (angiotensin-converting enzymes) in 15.2%, ACE inhibitors alone in 13.6%, and other combination preparations in 3.0%; the antihypertensive drug was unknown in 34.8% of the cases. Anticoagulation was carried out on 24.2% of patients because of other disease. Marcumar (10.6%) or NOACs (13.6%; new oral anticoagulants) were used for this purpose. Platelet inhibition was performed in 37.9% of cases. For this, ASA (30.3%; aspirin), clopidogrel and ASA combination preparation (7.6%), or clopidogrel (1.5%) were used.

### Case-specific documentation and communication

In 89.4% of cases, the outpatient consultation took place ≤ 10 days before the planned heart valve replacement. A completed consultation note from the TUM Klinikum Deutsches Herzzentrum München (DHM) was available in 83.3% of cases. A current doctor’s letter with a summary of the previous therapy, current medication, and the further procedure was available in 10.6% of cases. Current blood values, including coagulation parameters, were given to the patient at the outpatient presentation in 9.1% of cases.

The heart valve to be replaced was distributed as follows: 65.2% aortic valve, 27.3% mitral valve, 1.5% pulmonary valve, and in 6.0% of cases it was unclear. The subgroup analysis showed a gender distribution of the valve types as follows: Aortic valve 30 male and 13 female patients, mitral valve 15 male and three female patients, pulmonary valve one male and no female patients, and in a further three male patients the underlying valvular disease was not clear from the documents available and brought to the consultation. Patients with aortic valve disease had a median age of 72.5 years (35.6–88.4) and patients with mitral valve disease had a median age of 61.1 years (33.3–74.7) (*p* = 0,003). Arterial hypertension was present in 76.7% of patients with aortic valve disease and in 50% of patients with mitral valve disease. A consultation note was available for 86.0% of patients with aortic valve disease and 83.3% of patients with mitral valve disease. An additional physician’s letter was available for 14.0% of patients with aortic valve disease and 5.6% of patients with mitral valve disease.

Heart valve replacement was planned endovascularly in 27.3% of cases and openly in 9.1%. In 63.6% of cases, the type of valve replacement technique could not be determined from the available documentation and patient history. Valve stenosis was present in 57.6% of patients and valve insufficiency in 33.3%. In 9.1%, the underlying valve condition was not apparent from the documents provided for consultation. The severity of the valvular disease was distributed between grade III in 18.2%, grade IV in 16.7%, and unclear grading in 65.1% of patients.

### Clinical and radiological dental status

In 63.6% of cases, the patients stated that they went to the dentist regularly. According to the documentation, 1.5% of patients had very good oral hygiene, 59.1% good, 37.9% moderate, and 1.5% very poor. Some 80.3% had full or partial-dentures, with 48.5% having a crown restoration, 25.8% a bridge restoration, and 7.6% a prosthesis. Of the patients, 92.4% had tooth-preserving measures, 86.4% had a filling, and 74.2% a root canal filling. In the subgroup analysis, 48.4% of patients with aortic valve disease and 77.8% of patients with mitral valve disease reported regular visits to the dentist (*p* = 0.144). Thus, 16.3% of patients with aortic valve disease and 44.4% of patients with mitral valve disease had full dentition (*p* = 0.021). A further comparison of the subgroup analysis with regard to the clinical and radiological parameters is shown in Table 1.

**Table 1:** Subgroup analysis of clinical and radiological findings in patients with aortic (*n* = 43) or mitral (*n* = 18) valve disease

	Heart valve		
	Aortic valve	Mitral valve	*p*-value*
Pocket probing depth > 4 mm	58.1%	16.7%	0.002
Dental restoration	90.7%	94.4%	0.629
Dental root filling	71.1%	66.7%	0.750
Full or partial Dentures	83.7%	66.7%	0.141
Dental implant	7.0%	16.7%	0.250
OPG caries	58.1%	22.2%	0.009
OPG apical osteolysis	51.2%	16.7%	0.011
OPG dental root remnants	32.6%	5.6%	0.030

OPG = orthopantomogram

* Mann–Whitney U test

The maximum pocket probing depth was ≤ 4 mm for 56.0% of patients. The maximum tooth loosening of the examined patients was distributed as follows: grade 0 in 43.9%, grade I in 31.8%, and grade II in 24.2%. The overall DMFT index of the 66 patients was 19.0 [6–28]. In the subgroup analysis, the DMFT index was 20.0 [10–28] in patients with aortic valve disease and 18.5 [6–8] in patients with mitral valve disease (*p* = 0.209). The DMFT index was 20.0 [12–28] and 17.5 [12–28] (*p* = 0.304) in the comparison of patients with and without dental restoration. In the bivariate two-sided correlation analysis according to Spearman, there was a significant but weak negative correlation observed between the DMFT index and heart valve disease (ρ = −0.277; *p* = 0.029), indicating a limited inverse association between the variables. There was no significant correlation between the DMFT index and the indicated need for action (ρ = −0.032; *p* = 0.806) or between the DMFT index and a dental restoration (ρ = 0.171; *p* = 0.348).

### Need for oral and maxillofacial surgery and course of treatment

In 48.5% (*n* = 32) of cases, there was an indication for oral and maxillofacial surgery. The distribution of the two most common valves involved was 60.5% in patients with aortic valve disease and 22.2% in patients with mitral valve disease (*p* = 0.007). Further therapy was subsequently carried out in only 75.0% (*n* = 24) of cases, as a quarter of the patients refused necessary therapy after extensive information about the procedure and the associated risks and potential consequences of not undergoing the indicated procedure. In a further 18.8% (*n* = 6), the indicated and prepared and organized operation was canceled by the DHM itself at short notice on the day of the operation. Two patients wished to have their teeth restored by their own dentist. Thus, twelve patients with aortic valve disease and four patients with mitral valve disease underwent dental rehabilitation prior to valve replacement. The indicated procedure was performed a median of 2 days after outpatient presentation (0–10) and lasted a median of 60 min (15–90). A median of three teeth [1–10] were extracted or osteotomized during this operation time. In patients with mitral valve involvement, only one tooth was extracted or osteotomized in three cases and more than one tooth was extracted or osteotomized in one case.

No complications in terms of postoperative bleeding, wound dehiscence, or abscess formation were documented in the postoperative course for any case.

## Discussion

Based on the results of the meta-analysis by Talha et al., which included several countries with nationwide databases, the assumption is supported that the observed escalation in the incidence of IE is correct and is probably due to several factors, which include: [1] improvements in diagnosis; [2] changes in epidemiology and associated risk factors; [3] restrictions on the use of antibiotic prophylaxis promulgated through updated versions of guidelines; and [4] improvements and increased vigilance in coding practices and thus overall data collection and transparency [21]. Another contributing factor to the absolute increase of IE cases may be the rising number of heart valve replacements, particularly through minimal invasive, catheter-supported technique. This procedure allows the treatment of older and often more frail patients, who may consequently be at higher risk for IE development [11, 12]. Approximately half of post-TAVI PVE cases are healthcare-associated – more than twice as many as in post-SAVR PVE [15]. The increased exposure of this patient group to procedures associated with a risk of bacteremia may play a significant role in this higher incidence. For this reason, procedures with a high risk of bacteremia should always be strictly risk-stratified or proactively avoided through timely and appropriate dental focus clearance.

In the last decade, several studies have been published in the areas of the risk of adverse effects of antibiotic prophylaxis, the cost-effectiveness of antibiotic prophylaxis, the relationship between invasive dental procedures and the development of IE, the effectiveness of antibiotic prophylaxis, and the importance of good oral hygiene [25]. A recent study by Thornhill et al. analyzed the Hospital Episode Statistics database in the United Kingdom, which records all hospital admissions [26]. The specific patient histories of included patients were searched for intervention codes for the known entry points of IE development for each 30-day period over 15 months prior to IE-related hospitalization. The analysis revealed a significant association with IE after implantation of pacemakers/defibrillators (OR: 1.54, 95% CI 1.27–1.85; *p* < 0.001), extraction/surgical tooth removal (OR: 2.14, 95% CI 1.22–0.76; *p* = 0.047), endoscopic procedures on the upper (OR: 1.58, 95% CI 1.34–1.85; *p* < 0.001) and lower gastrointestinal tract (OR: 1.66, 95% CI 1.35–2.04; *p* < 0.001), and bone marrow biopsies (OR: 1.76, 95% CI 1.16–2.69; *p* = 0.039) [26]. The absolute risk was highest among individuals at high risk of IE who underwent tooth extraction/surgical tooth removal (49.5 per 100,000 procedures).

### Dental status prior to heart valve replacement

The pathogen spectrum varies depending on the pathogenesis. Early prosthetic endocarditis (within the first twelve months post-implantation [27]) is caused by direct intraoperative contamination or by hematogenous spread to the valve in the first days to weeks after surgery. Infective endocarditis in the period 2–12 months after surgery is secondary to late nosocomial or other out-of-hospital acquired infections [4]. The most common pathogens causing PVE are *Staphylococcus aureus*, *Streptococcus*, and *Enterococcus* [4, 6]. *Staphylococcus aureus* and *enterococci* have been detected more frequently as pathogens in PVE after TAVI [10].

The literature indicates that poor oral hygiene increases the risk of IE in people at medium and high cardiac risk [20]. Consequently, a risk stratification is based on the fact that certain cardiac conditions predispose patients to IE, making it more appropriate to treat or extract a critical tooth in these patients compared to those with a lower IE risk profile [25]. Further, Silvestre et al. observed in a prospective study significant differences in bacterial plaque index and probing depths between patients scheduled for heart valve replacement and controls, with higher scores in the patients with valve disease [22]. Tubiana et al. described after a database analysis and simulation that there was no statistically significant difference in the rate of oral *Streptococcus* after an invasive dental procedure without antibiotic prophylaxis compared to the period without exposure (in their cohort, 25% of invasive dental procedures were performed without antibiotic prophylaxis, contrary to national recommendations) [28]. These findings provided, for the first time in a large population size, an estimate of the incidence of PVE caused by oral *streptococci* in relation to everyday bacteremia (e.g., from oral hygiene habits such as brushing, using toothpicks, flossing, or chewing) in unexposed periods. The microtrauma caused by these everyday activities has been found to trigger oral streptococcal bacteremia at a similar rate to invasive oral procedures for which antibiotic prophylaxis is recommended. This knowledge is important and can be easily transferred to our results, as only 16.3% and 44.4% of the patients with aortic or mitral valve disease, respectively, had full dentition. This generally indicates a reduced dental status, which is also reflected in the DMFT index of 19.0 [6–28] overall. In the subgroup analysis, the DMFT index was 20.0 [10–28] in patients with aortic valve disease and 18.5 [6–28] in patients with mitral valve disease (*p* = 0.209). Thus, the DMFT index was higher overall in the patients we included in the oral focus search before heart valve replacement than in the seniors of the 6th German Oral Health Study (DMS 6) with a DMFT index of 17.6 [29].The DMS 5 showed a DMFT index of 17.7 [30]. However, it should be noted that the data acquisition of the DMS 5 published in 2016 was in 2014 and the DMFT index in the DMS 4 published in 2005, for example, was still 22.1. This trend is encouraging and may indicate that national awareness campaigns focused on oral health promotion are yielding a beneficial effect at the population level.

### Need for dental-surgical action prior to a heart valve replacement

In our study, 48.5% of the patient cases presented for consultation indicated the need for oral and maxillofacial surgery. Patients with aortic valve disease (in 60.5%) required intervention significantly more often (*p* = 0.007) than patients with mitral valve disease (in 22.2%). Overall, patients with an aortic valve disease were significantly more likely to have an increased pocket probing depth of more than 4 mm (*p* = 0.002), caries (*p* = 0.009), apical osteolysis (*p* = 0.011), and dental root remnants (*p* = 0.030) in the OPG (Table 1). Based on these clinical and radiological findings, twelve patients with aortic valve disease and four patients with mitral valve disease underwent tooth resection prior to valve replacement at our department. A median of three teeth were extracted or osteotomized during this surgical period. A study by Schmalz et al. pointed out that seriously ill patients with heart disease or requiring dialysis or organ transplants in particular showed inadequate oral hygiene behavior [31]. This statement is consistent with our results, that patients with aortic valve disease – especially aortic valve stenosis, which has a poor prognosis if left untreated – had a poorer oral hygiene status and a significantly higher need for action. Nevertheless, due to the overall small sample size and the discrepancy in group sizes, we acknowledge that a definitive conclusion regarding a significant association between poor oral hygiene and the type of heart valve disease cannot be drawn in this study.

The surgical effort involved in cardiac patients – especially patients with an aortic valve disease – is reflected on the one hand in the more complex saliva-tight wound closure and on the other hand in the additional use of dressing plates (18.8%) and Spongostan (43.8%) in these cases. The latter measures were not necessary in patients with mitral valve disease. In the included cases, the extraction sockets had to be closed saliva-tight with a gingivoperiosteal flap according to Rehrmann for plastic coverage, which is surgically more complex and time-consuming. These additional measures (cost drivers), which are generally intended to counteract bleeding complications, ensured that none of the analyzed complications (bleeding, abscess, and wound healing disruption) occurred in the postoperative course.

These results are also relevant in the well-known context that even everyday oral procedures (tooth brushing, flossing) can lead to temporary bacteremia. Martins et al. quantified the incidence of bacteremia in their meta analysis as follows: by tooth extraction in 62–66% of cases, by scaling in 36–44%, by tooth brushing in 8–26%, and by flossing in 16% [32]. The highest incidence of bacteremia was measured after five minutes for all procedures, which decreased in the following 6–20 min and could no longer be determined after two hours. Given that everyday oral procedures are less likely to cause bacteremia but are far more common than invasive dental procedures, there is an emerging consensus that improving oral hygiene is as important, if not more important, for the prevention of IE than providing antibiotic prophylaxis to people at risk of IE undergoing invasive dental procedures [32].

### Timing, treatment process, and consequences

The timing of the patient consultation for dental restoration prior to heart valve replacement should ideally take place 30 days [23] and at least ten days [24] before the planned heart valve replacement, provided the patient’s general and cardiac health situation permits. Based on this, it is vital for the consultant to be well-informed about the patient’s case in a consultation for a heart valve replacement. However, our results showed that patients were most frequently presented less than ten days before the planned cardiac procedure. Dental restoration took place a median of 2 days (0–10) after outpatient presentation and thus demonstrates prompt implementation of the necessary dental restoration prior to heart valve replacement. In our department, clearance for invasive measures (radiotherapy in the head and neck area, administration of chemotherapy or immunotherapy, or initiation or resumption of antiresorptive medication) is predominantly given after completion of gingival healing, which is ten days.

The minimum required information includes a current blood count, specifically levels of hemoglobin, kidney and thyroid values, and coagulation parameters. Additionally, a comprehensive medical letter detailing secondary diseases, especially cardiological assessments, is necessary to assess the risk of developing IE before the heart valve replacement. Certain cardiac findings increase the risk of IE [25], prompting the need for prompt dental interventions, such as restoring or extracting critical teeth, in patients with higher IE risk profiles. In a substantial proportion of cases (63.6%), the exact treatment modality was not known to the dental examiner at the time of assessment. This highlights a significant gap in interdisciplinary information transfer, which may directly impact clinical decision-making. To address this, barrier-free communication between cardiologists, cardiac surgeons, and the treating dentist or oral and maxillofacial surgeon must be established and strengthened. This would help avoid time-consuming follow-up queries and instead allow resources to be directed toward improving patient care. Gathering and assessing this information aids in effective risk stratification and decision-making in the context of heart valve replacement surgeries. In this study, the content quality of the necessary information transfer was primarily based on a consultation form filled out manually by the DHM, which was available in 83.3% of cases. A current doctor’s letter with a summary of the general medical history and illnesses – in order to be able to classify the risk as described above, i.e., into low, moderate, or high – including previous therapy, current medication, and the further procedure was available in only 10.6% of the patients consulted. Current blood values, including coagulation parameters, were given to the patient at the outpatient presentation in 9.1% of cases. In the same number of cases, the underlying valve condition, and in 65.1%, the grading of the valve condition was not clear from the documents provided for the consultation. If the relevant medical records are not accessible through a standardized IT solution, there should be clear and transparent communication with designated patient documentation. This facilitates the necessary educational discussion between the cardiologist, dentist, and patient to prevent the risk of IE or PVE. A correct antibiotic prophylaxis strategy as well as a dental restoration plan are a prerequisite to optimize the prevention of viridans group streptococcal IE/PVE through various synergistic approaches. Furthermore, it reduces the unnecessary administrative activity of determining the missing findings and can prevent duplicate examinations (e.g., blood tests, ECG). In addition to cost savings, this would significantly increase the time spent on patient care, reduce waiting times in the waiting room, and increase patient satisfaction.

Thornhill et al. analyzed a large cohort study of 7,951,972 commercial/Medicaid enrollees in the United States [23]. They recorded 3,774 hospitalizations for IE (475 cases/million), of which 34.2% were at high risk, 22.0% at moderate risk, and 43.8% at low/unknown risk of IE. The overall adjusted incidence of IE within 30 days of a dental procedure was 467.6 (high risk), 24.2 (moderate risk), and 3.8 (low/unknown risk) per million procedures. A sub-analysis of patients at high risk of IE showed an increased risk of developing IE especially after extractions (OR: 9.22; 95% CI: 5.54–15.88; *p* < 0.0001) and other oral surgery procedures (OR: 20.18; 95% CI: 11.22–36.74; *p* < 0.0001). Consequently, coverage with guideline-compliant antibiotic prophylaxis in the context of invasive dental procedures, which only occurred in 34.6% of patients, was associated with a significant reduction in the risk of IE in this patient group (OR: 0.38; 95% CI: 0.22–0.62; *p* = 0.002). These findings underline the real risk of IE during invasive dental procedures, especially extractions, in patients with a high risk of IE, so adequate knowledge of the patient history is essential.

However, only 56.2% (*n* = 18) of the patients in our study received the indicated dental restoration, as eight patients canceled the indicated dental restoration before heart valve replacement and a further six cases were canceled by the DHM at short notice on the day of surgery. Additionally, 25.0% of patients refused the necessary surgical tooth restoration in the form of an extraction or osteotomy, which is almost as high as the percentage that do not receive antibiotic prophylaxis according to guidelines, as described by others [23]. These patients therefore run an increased risk of developing PVE, despite being extensively informed.

### Future perspectives

In this patient group with an increased risk of IE, in addition to regular dental checkups, awareness should be raised through professional dental cleaning [20]. But in a survey on adherence for example, Ziebolz et al. concluded that the simple recommendation to visit the dentist is apparently insufficient to achieve adequate dental and periodontal treatment in patients with severe heart disease [33]. In a review by Østergaard et al., the authors described that PVE was found in 74.5% of cases analyzed within the first year after heart valve replacement [27]. After heart valve replacement, it is recommended to provide oral hygiene instructions, additional advice on oral care, and regular professional tooth cleaning for a period lasting from three to twelve months [27, 28]. Collaboration between dentists and other healthcare professionals is crucial, with motivating factors like recall bonuses and increased frequency of cleaning for high-risk patients. Verbal or visual advice alone is deemed insufficient for preventing infective endocarditis or prosthetic valve endocarditis [28, 33]. To address information gaps between healthcare providers and patients, organizational improvements and enhanced communication efforts are necessary. This interdisciplinary approach focuses on the importance of postoperative dental care for patients who have undergone heart valve replacement surgery.

In addition to frailty, another well-known problem is that ageing can have further negative effects on tooth structure, salivary flow, chewing and swallowing mechanisms (e.g., presbyphagia), the oral microbiome, and oropharyngeal sensitivity [34, 35]. Logistically, it will therefore be a challenge in the future for health insurance companies and those treating patients with TAVI due to the demographic trends of a growing but also older patient group, characterized by more cardiovascular comorbidities and an increased frailty index [11, 12]. Therefore, interdisciplinary care programs with dentists and oral and maxillofacial surgeons and the use of oral health promotion approaches are recommended.

### Limitations

This study is a purely retrospective analysis that aimed to investigate the general need for surgical intervention prior to heart valve replacement, with the intention of drawing clear attention to the importance of oral hygiene and the need for vigilant interdisciplinary cooperation. A follow-up study would be both valuable and interesting in order to further examine the impact of dental treatment – as well as the consequences of refusing such treatment – prior to heart valve replacement. Further, this retrospective study design does not allow us to draw any conclusions about the impact of the DMFT index on the clinical course or prognosis of the affected patients. This finding would also be of great interest in a prospective follow-up study of this historical cohort, which is currently being planned and may provide further valuable insights.

## Conclusions

This study highlights the frequent need for dental-surgical intervention in patients prior to heart valve replacement, as well as significant shortcomings in the consultation process – particularly due to communication gaps between disciplines and inconsistent patient compliance. Action is crucial, especially considering the proven significance of oral hygiene. To reduce the risk of prosthetic valve endocarditis, future efforts should focus on improving interdisciplinary education and coordination as well as ensuring timely, structured preoperative dental assessment.

## Data Availability

No datasets were generated or analysed during the current study.
